# Supplementary material for: Polar growth factor PgfA regulates polar peptidoglycan synthesis as well as mycolate synthesis in Mycobacterium smegmatis
Source: J Bacteriol. 2026 Jul 14;208(8):e00269-26. doi: 10.1128/jb.00269-26 (PMC13492138; doi:10.1128/jb.00269-26)
Supplement: Supplementary file 1 [file jb.00269-26-s0001.pdf]

Supplemental Table 1. Strain list.

Strain number	Genotype	Source	Figure panel
1278	Wild-type mc2155		Figure 4, 3B-G, 5
3433	mc^2155 L5::Pnative-Msm PgfA-mRFP-myc-nuoR ΔpgfA::zeoR	Rego Lab(1)	Figure 1, 3A
3438	mc^2155/ L5::pTetO-Msm PgfA-myc	Rego Lab(1)	Figure 3B-G, 5
3525	mc^2155 L5::pTetO-WT PgfA-mRFP-myc-nuoR		Figure 3A
3816	mc^2155/ L5::pMSMEG_0317 (pgfA).sgRNA		Figure 2

Supplemental Table 2. Plasmid list.

Strain number	Genotype	Source	Used in strain	Figure panel
3439	Dh5α/ pL5-pTetO-Msm PgfA-myc-nuo	Rego Lab(1)	3438	Figure 3B-G, 5
3806	Dh5α/ pMSMEG_0317 (pgfA).sgRNA)	MSRdb(2)	3816	Figure 2
3506	Top10/ pL5-pTetO-Msm WT PgfA-mRFP-myc		3525	Figure 3A

Supplemental Table 3. Primer list.

Strain number	Feature	Primer sequence
3506	Top10/ pL5-pTetO-Msm WT PgfA-mRFP-myc	Fwd:<br>AGATTCGCCGCCCGAAATGAGCACGATCCGCATGCTTAATTAAGAAGGAGATATACTAttgAACCGCGCTGTGGCGCTGCGT<br>Add mRFP to FL PgfA:<br>CACCGGACCGACCGATCggcggctcgGCCTCCTCCGAGGAC<br>Add mRFP to FL PgfA:<br>ttgatgaCGTCCTCGGAGGAGGCcgagccgccGATCGGTCGGTCCGG<br>Rev:<br>GGTCCCCAATTAATTAGCTAAAGCTTTCACAGGTCTTCCTCGCTGATCAGCTTCTGCTCGGCGCCGGTGGAGTGgcggccctcggcgc

Supplemental Figures

Supplemental Figure 1. PgfA-mRFP localization in log phase and carbon starvation

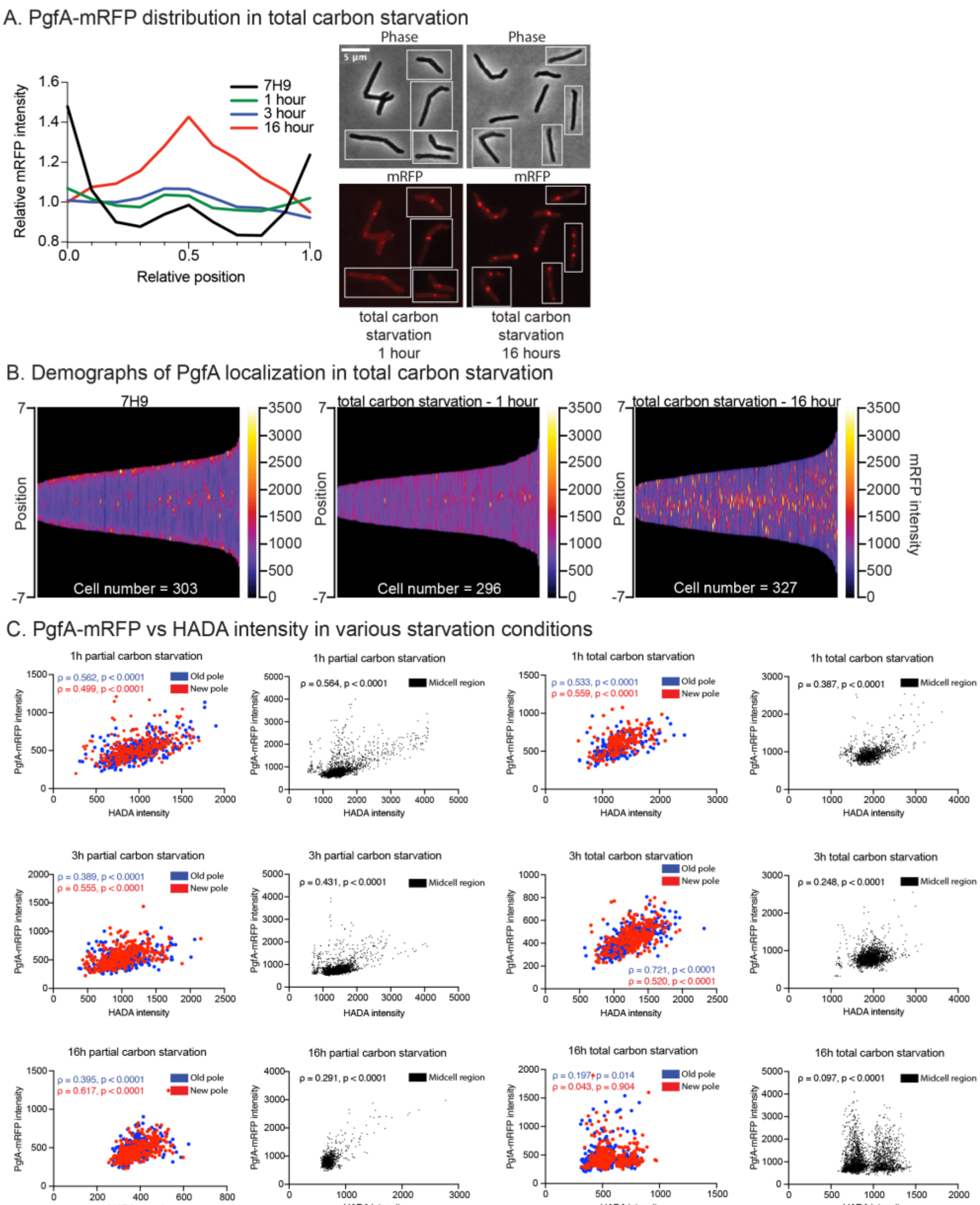

(A) Normalized mRFP fluorescence intensity profiles along the cell axis (0 = old pole, 1 = new pole; poles are assigned based on HADA intensity) during total carbon starvation. Cells were grown in 7H9 medium as a control (black) or resuspended in PBS supplemented with tyloxapol for 1 h (green), 3 h (blue), or 16 h (red). **Left**: fluorescence intensity profile. **Right**:

representative micrographs. Scale bar, 5 μm. Fluorescence intensity was normalized to the mean intensity of each cell, such that values above or below 1.0 indicate regions brighter or dimmer than the cell average. Each line represents the mean of n = 303 cells (7H9), 296 cells (1 h), and 327 cells (16 h).

(B) Demographs showing PgfA-mRFP intensity along the normalized cell axis for individual cells in 7H9, total carbon starvation at 1 h, and total carbon starvation at 16 h. Cells are sorted by length. Color scale indicates mRFP intensity. Cell numbers are indicated.

(C) Scatter plots of PgfA-mRFP versus HADA fluorescence intensities under partial (left two columns) and total (right two columns) carbon starvation at 1 h, 3 h, and 16 h. For each condition, polar mean intensities are shown with old pole (blue) and new pole (red) separately, and midcell region intensities are shown in black. Subcellular regions are defined as in Fig. 1A. Spearman rank correlation coefficients (ρ) and p-values are indicated.

Supplemental Figure 2. Raw peptidoglycan distribution in wild-type vs PgfA overexpression and antibiotic treatments

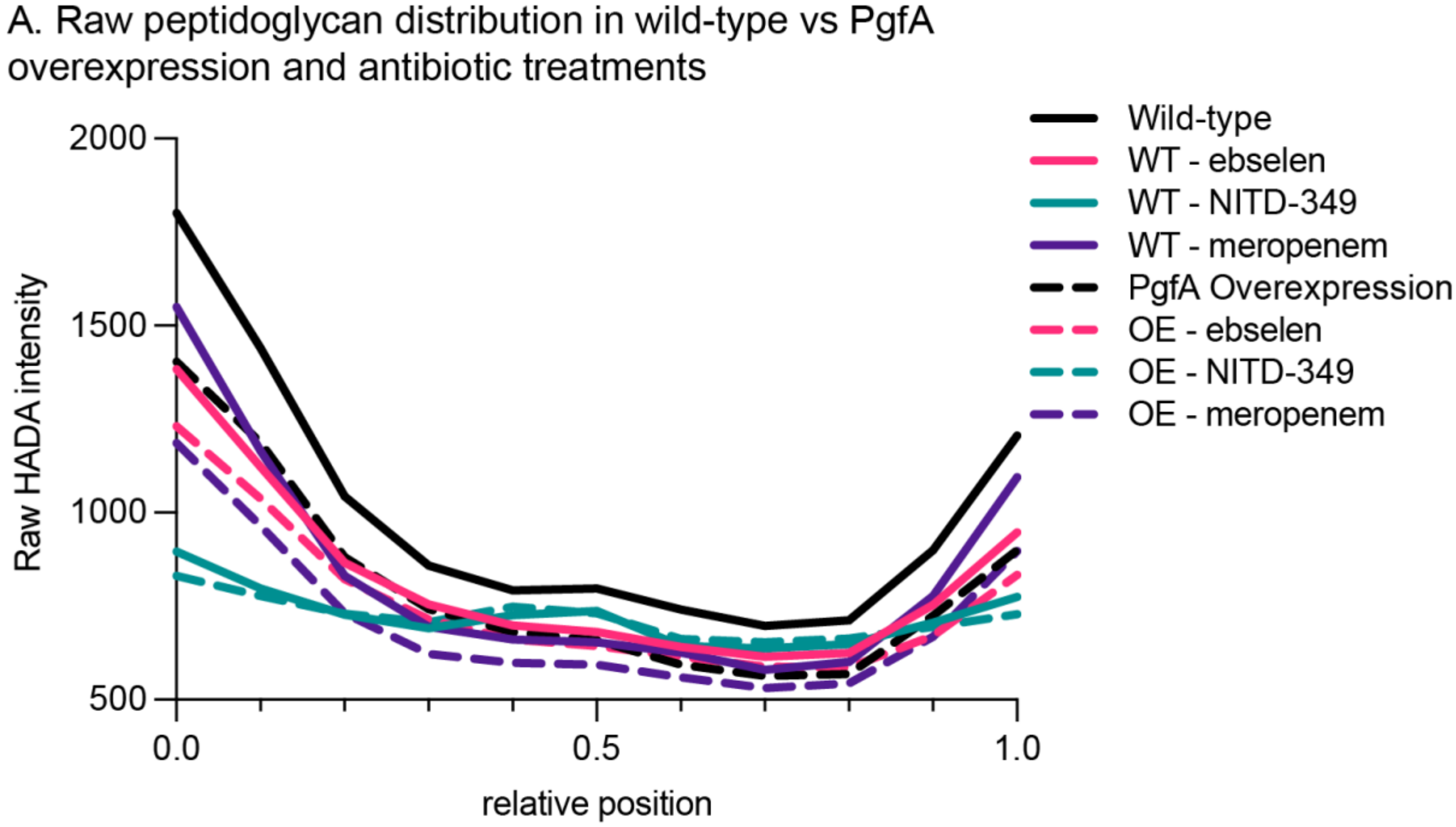

(A) Spatial distribution of HADA signal for wild-type and PgfA overexpression cells treated as in Figure 5. Raw HADA fluorescence intensity profiles along the cell axis (0 = old pole, 1 = new pole) for wild-type (solid lines) and PgfA overexpression (dashed lines) strains, untreated or treated with ebselen, NITD-349, or meropenem as in Fig. 5. Each line represents the mean of all cells per condition.

References:

1. Gupta KR, Gwin CM, Rahlwes KC, Biegas KJ, Wang C, Park JH, Liu J, Swarts BM, Morita YS, Rego EH. 2022. An essential periplasmic protein coordinates lipid trafficking and is required for asymmetric polar growth in mycobacteria. eLife 11:e80395.
2. Judd JA, Canestrari J, Clark R, Joseph A, Lapierre P, Lasek-Nesselquist E, Mir M, Palumbo M, Smith C, Stone M, Upadhyay A, Wirth SE, Dedrick RM, Meier CG, Russell DA, Dills A, Dove E, Kester J, Wolf ID, Zhu J, Rubin ER, Fortune S, Hatfull GF, Gray TA, Wade JT, Derbyshire KM. 2021. A Mycobacterial Systems Resource for the Research Community. mBio 12:e02401-20.

Supplemental Figure 3. Septal PgfA-mRFP and HADA colocalization analysis python code.

```
#!/usr/bin/env python3
"""
Septal Colocalization Analysis for GraphPad Prism
Analyzes mRFP and HADA colocalization at cell septa (midcell, position
0.4-0.6)
"""

import pandas as pd
import numpy as np
from scipy import stats

#
==========================================================================
======
# CONFIGURATION
#
==========================================================================
======
INPUT_FILE = '16hhard_both.xlsx'
SEPTAL_START = 0.4  # Start of septal region (normalized position)
SEPTAL_END = 0.6    # End of septal region (normalized position)
PEAK_DISTANCE_THRESHOLD = 0.1  # Consider peaks colocalized if within
this distance

#
==========================================================================
======
# READ DATA
#
==========================================================================
======
print("=" * 80)
print("SEPTAL COLOCALIZATION ANALYSIS")
print("=" * 80)

df = pd.read_excel(INPUT_FILE)

# Clean up column names
df = df.rename(columns={
    'CELL ID': 'Cell_ID_HADA',
    'X - HADA': 'Position_HADA',
    'Y - HADA': 'HADA_Intensity',
    'CELL ID.1': 'Cell_ID_mRFP',
    'X - pgfa': 'Position_mRFP',
    'Y - pgfa': 'mRFP_Intensity'
})

# Use HADA position and cell ID (they should be identical)
df['Position'] = df['Position_HADA']
df['Cell_ID'] = df['Cell_ID_HADA']
```

```
print(f"\nTotal datapoints: {len(df)}")
print(f"Unique cells: {df['Cell_ID'].nunique()}")

#
==========================================================================
======
# FILTER FOR SEPTAL REGION (0.4 - 0.6)
#
==========================================================================
======
septal_df = df[(df['Position'] >= SEPTAL_START) & (df['Position'] <=
SEPTAL_END)].copy()

print(f"\nSeptal datapoints (positions {SEPTAL_START}-{SEPTAL_END}):
{len(septal_df)}")
print(f"Cells with septal data: {septal_df['Cell_ID'].nunique()}")

#
==========================================================================
======
# CALCULATE HADA THRESHOLD
#
==========================================================================
======
# Define HADA-positive as mean + 1 SD
hada_mean = septal_df['HADA_Intensity'].mean()
hada_std = septal_df['HADA_Intensity'].std()
hada_threshold = hada_mean + hada_std

septal_df['HADA_above_threshold'] = septal_df['HADA_Intensity'] >
hada_threshold

print(f"\nHADA threshold: {hada_threshold:.2f} (mean + 1 SD)")
print(f"Datapoints above threshold:
{septal_df['HADA_above_threshold'].sum()}")

#
==========================================================================
======
# PER-CELL ANALYSIS
#
==========================================================================
======
print("\n" + "=" * 80)
print("CALCULATING PER-CELL METRICS")
print("=" * 80)

cell_metrics_list = []
```

```
for cell_id, cell_data in septal_df.groupby('Cell_ID'):
    if len(cell_data) < 2:  # Need at least 2 points for correlation
        continue

    # Pearson correlation
    pearson_r =
cell_data['HADA_Intensity'].corr(cell_data['mRFP_Intensity'])
    r_squared = pearson_r ** 2 if not pd.isna(pearson_r) else 0

    # Find peak positions
    hada_peak_idx = cell_data['HADA_Intensity'].idxmax()
    mrfp_peak_idx = cell_data['mRFP_Intensity'].idxmax()

    hada_peak_pos = cell_data.loc[hada_peak_idx, 'Position']
    mrfp_peak_pos = cell_data.loc[mrfp_peak_idx, 'Position']

    # Peak distance
    peak_distance = abs(hada_peak_pos - mrfp_peak_pos)
    peaks_colocalized = peak_distance < PEAK_DISTANCE_THRESHOLD

    # mRFP enrichment at HADA-positive positions
    hada_positive = cell_data[cell_data['HADA_above_threshold']]
    hada_negative = cell_data[~cell_data['HADA_above_threshold']]

    if len(hada_positive) > 0 and len(hada_negative) > 0:
        mrfp_at_hada_peaks = hada_positive['mRFP_Intensity'].mean()
        mrfp_at_hada_neg = hada_negative['mRFP_Intensity'].mean()
        enrichment = mrfp_at_hada_peaks / mrfp_at_hada_neg if
mrfp_at_hada_neg > 0 else np.nan
    else:
        enrichment = np.nan

    cell_metrics_list.append({
        'Cell_ID': cell_id,
        'Pearson_r': pearson_r,
        'R_squared': r_squared,
        'HADA_peak_position': hada_peak_pos,
        'mRFP_peak_position': mrfp_peak_pos,
        'Peak_distance': peak_distance,
        'Peaks_colocalized': peaks_colocalized,
        'mRFP_enrichment_at_HADA_peaks': enrichment,
        'N_datapoints_in_septum': len(cell_data)
    })

cell_metrics_df = pd.DataFrame(cell_metrics_list)

#
=======================================================================
======
# GLOBAL CORRELATION (ALL SEPTAL POINTS COMBINED)
```

```
#
==========================================================================
======
global_pearson_r =
septal_df['HADA_Intensity'].corr(septal_df['mRFP_Intensity'])
global_r_squared = global_pearson_r ** 2

# Linear regression for plotting
slope, intercept, r_value, p_value, std_err = stats.linregress(
    septal_df['HADA_Intensity'],
    septal_df['mRFP_Intensity']
)

print(f"\nGlobal correlation (all septal datapoints):")
print(f"  Pearson r = {global_pearson_r:.3f}")
print(f"  R² = {global_r_squared:.3f}")
print(f"  p-value = {p_value:.2e}")
print(f"  Linear fit: y = {slope:.3f}x + {intercept:.2f}")

#
==========================================================================
======
# SUMMARY STATISTICS
#
==========================================================================
======
print("\n" + "=" * 80)
print("SUMMARY STATISTICS")
print("=" * 80)

# Remove NaN values for statistics
valid_r2 = cell_metrics_df['R_squared'].dropna()
valid_enrichment =
cell_metrics_df['mRFP_enrichment_at_HADA_peaks'].dropna()

print(f"\nPer-cell R² distribution:")
print(f"  Mean R² = {valid_r2.mean():.3f} ± {valid_r2.std():.3f}
(SD)")
print(f"  Median R² = {valid_r2.median():.3f}")
print(f"  Range: {valid_r2.min():.3f} to {valid_r2.max():.3f}")
print(f"  N cells = {len(valid_r2)}")

colocalized_count = cell_metrics_df['Peaks_colocalized'].sum()
total_cells = len(cell_metrics_df)
colocalized_pct = 100 * colocalized_count / total_cells

print(f"\nPeak colocalization:")
print(f"  Cells with colocalized peaks: {colocalized_count}/
{total_cells} ({colocalized_pct:.1f}%)")
print(f"  Mean peak distance:
```

```
{cell_metrics_df['Peak_distance'].mean():.3f} ±
{cell_metrics_df['Peak_distance'].std():.3f}")

if len(valid_enrichment) > 0:
    print(f"\nmRFP enrichment at HADA-positive regions:")
    print(f"  Mean enrichment: {valid_enrichment.mean():.2f}-fold")
    print(f"  Median enrichment: {valid_enrichment.median():.2f}-
fold")

#
======================================================================
======
# EXPORT FOR GRAPHPAD PRISM
#
======================================================================
======
print("\n" + "=" * 80)
print("EXPORTING DATA FOR PRISM")
print("=" * 80)

# 1. All septal datapoints for scatter plot
output1 = septal_df[['Cell_ID', 'Position', 'HADA_Intensity',
'mRFP_Intensity', 'HADA_above_threshold']].copy()
output1.to_csv('prism_septal_all_points.csv', index=False)
print("\n1. prism_septal_all_points.csv")
print("   → Use for scatter plot (HADA vs mRFP)")
print("   → X: HADA_Intensity, Y: mRFP_Intensity")

# 2. Per-cell metrics
cell_metrics_df.to_csv('prism_per_cell_metrics.csv', index=False)
print("\n2. prism_per_cell_metrics.csv")
print("   → Use for R² distribution, peak colocalization stats")
print("   → Plot R_squared as violin/column plot")

# 3. Summary statistics table
summary_stats = pd.DataFrame({
    'Metric': [
        'Global R²',
        'Global Pearson r',
        'Mean per-cell R²',
        'SD per-cell R²',
        '% cells with colocalized peaks',
        'Mean peak distance',
        'N cells analyzed'
    ],
    'Value': [
        f"{global_r_squared:.3f}",
        f"{global_pearson_r:.3f}",
        f"{valid_r2.mean():.3f}",
        f"{valid_r2.std():.3f}",
```

```
        f"{colocalized_pct:.1f}%",
        f"{cell_metrics_df['Peak_distance'].mean():.3f}",
        f"{len(cell_metrics_df)}"
    ]
})
summary_stats.to_csv('prism_summary_stats.csv', index=False)
print("\n3. prism_summary_stats.csv")
print("   → Summary table for your paper")

# 4. Regression data for Prism
regression_data = pd.DataFrame({
    'HADA_Intensity': septal_df['HADA_Intensity'],
    'mRFP_Intensity': septal_df['mRFP_Intensity'],
    'Fitted_line': slope * septal_df['HADA_Intensity'] + intercept
})
regression_data.to_csv('prism_regression_data.csv', index=False)
print("\n4. prism_regression_data.csv")
print("   → Use to plot regression line on scatter plot")

print("\n" + "=" * 80)
print("ANALYSIS COMPLETE!")
print("=" * 80)
print("\nTo use in GraphPad Prism:")
print("1. Import 'prism_septal_all_points.csv' for XY scatter plot")
print("2. Analyze → XY analysis → Linear regression (to get R²)")
print("3. Import 'prism_per_cell_metrics.csv' for per-cell
statistics")
print("4. Use 'prism_summary_stats.csv' for your methods/results")
```

Supplemental Figure 4. Batch polar correlation calculator python code.

```
#!/usr/bin/env python3
"""
Calculates Spearman correlations for old pole and new pole separately

INPUT: One Excel file with multiple sheets (one sheet per condition)
Each sheet should have columns:
  - Pole 1 P = old pole pgfA/mRFP intensity
  - Pole 1 H = old pole HADA intensity
  - Pole 2 P = new pole pgfA/mRFP intensity
  - Pole 2 H = new pole HADA intensity

OUTPUT: CSV file with correlation statistics for each condition (old
and new poles separate)
"""

import pandas as pd
import numpy as np
from scipy import stats

#
======================================================================
======
# CONFIGURATION - EDIT THIS
#
======================================================================
======

INPUT_FILE = 'pole_intensity_data.xlsx'  # <- Your Excel file name

# Column names in your Excel sheets. Change as necessary for your
gene/condition.
# Make sure to change column names throughout the rest of the code!
OLD_POLE_MRFP = 'Pole 1 P'
OLD_POLE_HADA = 'Pole 1 H'
NEW_POLE_MRFP = 'Pole 2 P'
NEW_POLE_HADA = 'Pole 2 H'

#
======================================================================
======
# EDIT CAREFULLY BELOW THIS LINE
#
======================================================================
======

print("=" * 80)
print("BATCH POLAR CORRELATION ANALYSIS")
print("=" * 80)
print(f"\nInput file: {INPUT_FILE}")
```

```python
# Read Excel file
excel_file = pd.ExcelFile(INPUT_FILE)
sheet_names = excel_file.sheet_names

print(f"Found {len(sheet_names)} sheets: {sheet_names}")
print()

results = []

for sheet_name in sheet_names:
    print(f"Processing: {sheet_name}")

    # Read the sheet
    df = pd.read_excel(excel_file, sheet_name=sheet_name)

    print(f"   Rows: {len(df)}")

    #
======================================================================
==
    # OLD POLE (Pole 1)
    #
======================================================================
==

    old_pole_mrfp = df[OLD_POLE_MRFP].values
    old_pole_hada = df[OLD_POLE_HADA].values

    # Remove NaN
    mask_old = ~(np.isnan(old_pole_mrfp) | np.isnan(old_pole_hada))
    old_pole_mrfp = old_pole_mrfp[mask_old]
    old_pole_hada = old_pole_hada[mask_old]

    if len(old_pole_mrfp) >= 3:
        # Pearson
        pearson_r_old, pearson_p_old = stats.pearsonr(old_pole_hada,
old_pole_mrfp)

        # Spearman
        spearman_r_old, spearman_p_old =
stats.spearmanr(old_pole_hada, old_pole_mrfp)

        # Kendall
        kendall_tau_old, kendall_p_old =
stats.kendalltau(old_pole_hada, old_pole_mrfp)

        print(f"   📊 OLD POLE:")
        print(f"      n = {len(old_pole_mrfp)}")
        print(f"      Pearson:  r = {pearson_r_old:.4f}, r² =
```

```
{pearson_r_old**2:.4f}")
        print(f"      Spearman: ρ = {spearman_r_old:.4f}, ρ² =
{spearman_r_old**2:.4f}")
        print(f"      Kendall:  τ = {kendall_tau_old:.4f}")

        results.append({
            'Condition': sheet_name,
            'Pole': 'Old',
            'n_cells': len(old_pole_mrfp),
            'Pearson_r': pearson_r_old,
            'Pearson_r_squared': pearson_r_old ** 2,
            'Pearson_p': pearson_p_old,
            'Spearman_rho': spearman_r_old,
            'Spearman_rho_squared': spearman_r_old ** 2,
            'Spearman_p': spearman_p_old,
            'Kendall_tau': kendall_tau_old,
            'Kendall_p': kendall_p_old
        })
    else:
        print(f"   ❌ OLD POLE: Not enough data ({len(old_pole_mrfp)}
cells)")

    #
======================================================================
==
    # NEW POLE (Pole 2)
    #
======================================================================
==

    new_pole_mrfp = df[NEW_POLE_MRFP].values
    new_pole_hada = df[NEW_POLE_HADA].values

    # Remove NaN
    mask_new = ~(np.isnan(new_pole_mrfp) | np.isnan(new_pole_hada))
    new_pole_mrfp = new_pole_mrfp[mask_new]
    new_pole_hada = new_pole_hada[mask_new]

    if len(new_pole_mrfp) >= 3:
        # Pearson
        pearson_r_new, pearson_p_new = stats.pearsonr(new_pole_hada,
new_pole_mrfp)

        # Spearman
        spearman_r_new, spearman_p_new =
stats.spearmanr(new_pole_hada, new_pole_mrfp)

        # Kendall
        kendall_tau_new, kendall_p_new =
stats.kendalltau(new_pole_hada, new_pole_mrfp)
```

```
        print(f"    📊 NEW POLE:")
        print(f"      n = {len(new_pole_mrfp)}")
        print(f"      Pearson:  r = {pearson_r_new:.4f}, r² =
{pearson_r_new**2:.4f}")
        print(f"      Spearman: ρ = {spearman_r_new:.4f}, ρ² =
{spearman_r_new**2:.4f}")
        print(f"      Kendall:  τ = {kendall_tau_new:.4f}")

        results.append({
            'Condition': sheet_name,
            'Pole': 'New',
            'n_cells': len(new_pole_mrfp),
            'Pearson_r': pearson_r_new,
            'Pearson_r_squared': pearson_r_new ** 2,
            'Pearson_p': pearson_p_new,
            'Spearman_rho': spearman_r_new,
            'Spearman_rho_squared': spearman_r_new ** 2,
            'Spearman_p': spearman_p_new,
            'Kendall_tau': kendall_tau_new,
            'Kendall_p': kendall_p_new
        })
    else:
        print(f"    ❌ NEW POLE: Not enough data ({len(new_pole_mrfp)}
cells)")

    print()

#
======================================================================
======
# SAVE RESULTS
#
======================================================================
======

if len(results) == 0:
    print("❌ No data was successfully analyzed!")
    exit(1)

results_df = pd.DataFrame(results)

# Save to CSV
output_file = 'batch_polar_correlations.csv'
results_df.to_csv(output_file, index=False)

print("=" * 80)
print("RESULTS SUMMARY")
print("=" * 80)
print()
```

```
print(results_df[['Condition', 'Pole', 'n_cells', 'Pearson_r_squared',
                  'Spearman_rho',
'Spearman_rho_squared']].to_string(index=False))

print("\n" + "=" * 80)
print("SAVED TO: " + output_file)
print("=" * 80)

#
======================================================================
======
# CREATE FORMATTED OUTPUT FOR PAPER
#
======================================================================
======

print("\n" + "=" * 80)
print("FOR YOUR PAPER (copy-paste ready)")
print("=" * 80)

for condition in results_df['Condition'].unique():
    cond_data = results_df[results_df['Condition'] == condition]

    print(f"\n{condition}:")

    for _, row in cond_data.iterrows():
        pole = row['Pole']
        rho = row['Spearman_rho']
        p = row['Spearman_p']
        n = int(row['n_cells'])
        p_str = f"p = {p:.2e}" if p >= 0.0001 else "p < 0.0001"

        print(f"  {pole} pole: ρ = {rho:.3f}, {p_str}, n = {n}")

print("\n✅ ANALYSIS COMPLETE!")
print(f"✅ Successfully analyzed {len(results_df)//2} conditions × 2
poles = {len(results_df)} datasets")
print(f"✅ Results saved to: {output_file}")
```

Supplemental Figure 5. Batch septal correlation calculation python code.

```
#!/usr/bin/env python3
"""
Calculates Pearson, Spearman, and Kendall correlations for
multiple datasets

HOW TO USE:
1. Put all your Excel files in the same folder as this script
2. Edit the FILES_TO_ANALYZE list below (lines 18-26)
3. Run: python batch_correlations.py
4. Results will be saved to: batch_correlation_results.csv

REQUIREMENTS:
- Excel files must have columns containing 'X' or 'Position' (for cell
position 0-1)
- Excel files must have columns with 'HADA' and 'Y' (for HADA
intensity)
- Excel files must have columns with 'pgfa' or 'mRFP' and 'Y' (for
mRFP intensity)
"""

import pandas as pd
import numpy as np
from scipy import stats
import os

#
======================================================================
======
# EDIT THIS SECTION - ADD OR REMOVE FILES AS NEEDED
#
======================================================================
======

FILES_TO_ANALYZE = [
    # Format: ('Display Name', 'filename.xlsx')
    ('Control', 'ctrl_both.xlsx'),
    ('Experimental', 'experimental.xlsx'),
]

# Optional: Change septal region boundaries (default: 0.4-0.6)
SEPTAL_START = 0.4
SEPTAL_END = 0.6

#
======================================================================
======
# NO NEED TO EDIT BELOW THIS LINE
#
======================================================================
```

```
======

def find_column(df, keywords):
    """Find column matching any of the keywords (case insensitive)"""
    for col in df.columns:
        col_lower = col.lower()
        if all(kw.lower() in col_lower for kw in keywords):
            return col
    return None

def analyze_file(filename, display_name):
    """Analyze one file and return correlation statistics"""

    try:
        # Check if file exists
        if not os.path.exists(filename):
            print(f"   ❌ File not found: {filename}")
            return None

        # Read Excel file
        df = pd.read_excel(filename)

        # Find required columns. Change 'pgfa' to 'your gene name' or
'your fp'. Change in
        # subsequent sections to prevent errors
        pos_col = find_column(df, ['x']) or find_column(df,
['position'])
        hada_col = find_column(df, ['y', 'hada'])
        mrfp_col = find_column(df, ['y', 'pgfa']) or find_column(df,
['y', 'mrfp'])

        if not all([pos_col, hada_col, mrfp_col]):
            print(f"   ❌ Could not find required columns")
            print(f"      Available columns: {list(df.columns)}")
            return None

        print(f"   📁 Using: Position={pos_col}, HADA={hada_col},
mRFP={mrfp_col}")

        # Filter for septal region
        septal = df[(df[pos_col] >= SEPTAL_START) & (df[pos_col] <=
SEPTAL_END)]

        if len(septal) == 0:
            print(f"   ❌ No data in septal region ({SEPTAL_START}-
{SEPTAL_END})")
            return None

        # Extract intensities
        hada = septal[hada_col].values
```

```
        mrfp = septal[mrfp_col].values

        # Remove NaN
        mask = ~(np.isnan(hada) | np.isnan(mrfp))
        hada = hada[mask]
        mrfp = mrfp[mask]

        if len(hada) < 3:
            print(f"   ❌ Too few valid datapoints ({len(hada)})")
            return None

        print(f"   ✅ {len(hada)} valid septal datapoints")

        # Calculate correlations
        pearson_r, pearson_p = stats.pearsonr(hada, mrfp)
        spearman_r, spearman_p = stats.spearmanr(hada, mrfp)
        kendall_tau, kendall_p = stats.kendalltau(hada, mrfp)

        # Display results
        print(f"   📊 Pearson:  r = {pearson_r:.4f}, r² =
{pearson_r**2:.4f}")
        print(f"   📊 Spearman: ρ = {spearman_r:.4f}, ρ² =
{spearman_r**2:.4f}")
        print(f"   📊 Kendall:  τ = {kendall_tau:.4f}")

        return {
            'Condition': display_name,
            'Filename': filename,
            'n_datapoints': len(hada),
            'Pearson_r': pearson_r,
            'Pearson_r_squared': pearson_r ** 2,
            'Pearson_p': pearson_p,
            'Spearman_rho': spearman_r,
            'Spearman_rho_squared': spearman_r ** 2,
            'Spearman_p': spearman_p,
            'Kendall_tau': kendall_tau,
            'Kendall_p': kendall_p
        }

    except Exception as e:
        print(f"   ❌ Error analyzing {filename}: {str(e)}")
        return None

#
======================================================================
======
# MAIN ANALYSIS
#
======================================================================
======
```

```
print("=" * 80)
print("BATCH SPEARMAN CORRELATION ANALYSIS")
print("=" * 80)
print(f"\nAnalyzing septal region: {SEPTAL_START}-{SEPTAL_END}")
print(f"Number of files to analyze: {len(FILES_TO_ANALYZE)}")
print()

results = []

for display_name, filename in FILES_TO_ANALYZE:
    print(f"Processing: {display_name}")
    result = analyze_file(filename, display_name)
    if result is not None:
        results.append(result)
    print()

#
======================================================================
======
# SAVE RESULTS
#
======================================================================
======

if len(results) == 0:
    print("❌ No files were successfully analyzed!")
    print("Check that:")
    print("  1. Files exist in the same folder as this script")
    print("  2. Filenames in FILES_TO_ANALYZE are correct")
    print("  3. Files have the required columns (X, Y-HADA, Y-pgfa)")
    exit(1)

results_df = pd.DataFrame(results)

# Save to CSV
output_file = 'batch_correlation_results.csv'
results_df.to_csv(output_file, index=False)

print("=" * 80)
print("RESULTS SUMMARY")
print("=" * 80)
print()
print(results_df[['Condition', 'n_datapoints', 'Pearson_r_squared',
                  'Spearman_rho',
'Spearman_rho_squared']].to_string(index=False))

print("\n" + "=" * 80)
print("SAVED TO: " + output_file)
print("=" * 80)
```

```
# Create a summary for easy copy-paste into report
print("\n" + "=" * 80)
print("FOR YOUR REPORT (copy-paste ready)")
print("=" * 80)

print("\nSpearman correlation coefficients (septal region):")
for _, row in results_df.iterrows():
    rho = row['Spearman_rho']
    p = row['Spearman_p']
    n = int(row['n_datapoints'])
    p_str = f"p = {p:.2e}" if p >= 0.0001 else "p < 0.0001"
    print(f"  {row['Condition']:12s}: ρ = {rho:.3f}, {p_str}, n =
{n}")

print("\n ANALYSIS COMPLETE!")
print(f"Successfully analyzed {len(results)}/{len(FILES_TO_ANALYZE)}
files")
print(f"Results saved to: {output_file}")
```
